# The mitochondrial calcium uniporter: a new therapeutic target for Parkinson’s disease-related cardiac dysfunctions?

**DOI:** 10.6061/clinics/2020/e1299

**Published:** 2020-01-06

**Authors:** Fúlvio Alexandre Scorza, Francisco Sandro Menezes-Rodrigues, Efraín Olszewer, Paolo Ruggero Errante, José Gustavo Patrão Tavares, Carla Alessandra Scorza, Henrique Ballalai Ferraz, Josef Finsterer, Afonso Caricati-Neto

**Affiliations:** I Disciplina de Neurociencia, Escola Paulista de Medicina, Universidade Federal de Sao Paulo (UNIFESP), Sao Paulo, SP, BR; II Departamento de Farmacologia, Escola Paulista de Medicina, Universidade Federal de Sao Paulo (UNIFESP), Sao Paulo, SP, BR; III Fundacao de Apoio a Pesquisa e Estudos na Area de Saude (FAPES), Sao Paulo, SP, BR; IV Departamento de Neurologia. Escola Paulista de Medicina, Universidade Federal de Sao Paulo (UNIFESP), Sao Paulo, SP, BR; V Krankenanstalt Rudolfstiftung, Messerli Institute, Vienna, Austria

In 1817, the London surgeon and pharmacist James Parkinson published a 66-page-long booklet entitled “An Essay on the Shaking Palsy”, which contains the first detailed, clinical description of the shaking palsy, or “paralysis agitans”, which is currently known as Parkinson’s disease (PD) (1). Two important facts James Parkinson probably did not consider at that time include the scientific progress of neuroscience over the past two centuries and the changes in the world’s population aging since then (2). In fact, the world’s population is steadily becoming older. From 2015 to 2030, the number of individuals in the world aged ≥60 years is estimated to grow by 56%, from 901 million to 1.4 billion, and by 2050, it is estimated to be more than double the size of that of 2015, reaching approximately 2.1 billion (3). Unfortunately, aging is the main risk factor for major human diseases, such as neurological and cardiovascular disorders (4). Thus, the concluding remarks of the “Global Burden of Diseases, Injuries, and Risk Factors” (GBD) report are clear in stating that neurological disorders are a main cause of disability and death worldwide. Globally, the burden of neurological conditions has increased substantially over the past 25 years because populations are getting older (5).

PD affects millions of people globally, but there is no cure, and its prevalence will double by 2030 (6-8). Although PD is not considered a “malignant“ or even a “fatal” disease, mortality is not a negligible matter among patients with PD. Recently, we analyzed mortality in PD. Of the approximately 97,000 scientific articles on PD analyzed in our study, 1650 articles related to mortality in PD were found (9). Data from several well-designed studies suggest that mortality in PD patients is higher than that seen in the general population (9-12,14). A large prospective cohort study clearly demonstrated that mortality in PD is not increased in the first 5 years after onset but increases thereafter, with a relative risk of 3.5 after 10 years (12,13). The leading causes of death in PD are pneumonia and cardiovascular diseases (14,15). Approximately 60% of PD patients have cardiovascular disorders (9,15). These disorders are present in almost all stages of PD, and heart rate variability seems to be a key feature, becoming less variable before any motor symptoms suggest PD (9,15).

The neuroscientific community has recently recognized that an increasing number of PD patients has died suddenly and unexpectedly, referred to as “sudden unexpected death in Parkinson’s disease” (SUDPAR) (9,). SUDPAR has been defined as an unexpected death in a patient with PD without any satisfactory explanation for death as determined by autopsy studies (9,15-19). So far, a number of risk factors may be associated with SUDPAR, such as age at onset, duration of PD, sex, motor severity, and type and duration of drug therapy (polypharmacy) (9,10,16,17,20-22). Although sudden cardiac death rates range from 50 to 100 per 100,000 in the general population (9,15), the true incidence of SUDPAR is completely unknown. While the specific risk factors and mechanisms of SUDPAR are not fully understood, its prevention is crucial (9,15-19).

Considering that SUDPAR is a rare phenomenon, difficult to diagnose, and only rarely reported, it is a phenomenon that has attracted the interest of the neuroscientific community since the late 1970s (15). More recently, experimental and clinical evidence has suggested that autonomic and myocardial dysfunctions could directly be involved in SUDPAR (9,16-18,21,23-26). Some evidence suggests that autonomic dysfunctions are variable and caused by the deregulation of both the sympathetic and parasympathetic mechanisms involved in the neurogenic regulation of cardiac activity (15,22-24). Although sympathetic hypoactivity and parasympathetic hyperactivity have been associated with cardiac dysfunctions in PD (15,22-24), cellular and molecular mechanisms involved in these dysfunctions remain unclear.

PD-related cardiac dysfunction may manifest as ventricular arrhythmias due to the collapse of cardiac excitation-contraction coupling (CECC), primarily caused by persistent ionic deregulation in cardiomyocytes (9,15,17,22,24). This deregulation is mainly caused by the abnormal activity of proteins and cytoplasmic organelles involved in the precise adjustment of cytosolic Ca^2+^ concentration ([Ca^2+^]_c_) and energy production in cardiomyocytes, such as Ca^2+^ channels, Ca^2+^-ATPases, the sarcoplasmic reticulum (SR), and mitochondria (MIT) (24,27,28). In mammalian cardiomyocytes, the mitochondrial network occupies approximately 30% of the cell volume and accounts for approximately 95% of the cellular production of energy stored as adenosine triphosphate (ATP) molecules (27,28). MIT also play a key role in the contractile activity of these cells due to their involvement in Ca^2+^ homeostasis (27,28).

The heart rate depends on the electrical and mechanical properties of the myocardium, and these depend on CECC. When stimulated by the electrical impulses generated and transmitted by the specialized cardiac cells, the plasma membrane of cardiomyocytes is depolarized, allowing Ca^2+^ influx from the extracellular medium to the cytosol through L-type voltage-dependent Ca^2+^ channels (VDCCs) (27,28). This Ca^2+^ influx stimulates Ca^2+^-release (CICR) from the SR via ryanodine-sensitive Ca^2+^ channels (RyRs), generating a transient elevation in the [Ca^2+^]_c_ and consecutive activation of the myosin-actin contractile myofilaments. This transient elevation in [Ca^2+^]_c_ simultaneously increases the Ca^2+^ uptake by MIT and the Ca^2+^ concentration in the mitochondrial matrix ([Ca^2+^]_m_), which stimulates ATP production by the activation of the dehydrogenases in the tricarboxylic acid (TCA) cycle. To generate contractility for the ejection of blood from the heart, the activation of myosin by energy-stored ATP molecules is required to shift the head pulling on the actin filament and to shorten the sarcomere. The strength of myocardial contraction is directly related to the local Ca^2+^ concentration surrounding the myosin-actin myofilaments. Thus, the synchronization of [Ca^2+^]_c_ transients throughout the myocardium is crucial for synchronous cardiac contraction (27,28). However, the deregulation of [Ca^2+^]_c_ induces mechanical desynchrony, which induces cardiac arrhythmias (27,28). In some circumstances, these arrhythmias can be extremely severe or even fatal.

In mammalian cardiomyocytes, Ca^2+^ uptake by MIT is mainly mediated by the mitochondrial uniporter of Ca^2+^ (MUC), while its efflux is mainly mediated by the mitochondrial Na^+^/Ca^2+^-exchange channel (mNCE) (Figure 1). Thus, the functions of MIT strongly depend on the activity of the MUC and mNCE to maintain the dynamic equilibrium between the Ca^2+^ influx/efflux and [Ca^2+^]_m_ (27-30). However, pathophysiological processes that cause ionic deregulation, such as cardiac ischemia and reperfusion (IR) injury, produce sustained increases in [Ca^2+^]_c_ and [Ca^2+^]_m_, culminating in the collapse of the functions of MIT that dramatically affect ATP production (24,27-29). The collapse of MIT in cardiomyocytes compromises the functioning of ATP-dependent cellular processes, such as transmembrane transport of Ca^2+^, Na^+^ and K^+^, aggravating mechanical desynchrony and increasing the incidence of cardiac arrhythmias (27-29).

It has been shown that mutations in genes causing PD, such as PINK1, parkin, DJ-1, alpha-synuclein, and LRRK2, cause mitochondrial dysfunctions, which is one of the reasons why they are called mitochondrial nigropathies (31). Mitochondrial disorders associated with PD may also result from oxidative stress or exogenous toxins (31). To date, there are no consistent data in the scientific literature to establish whether the risk of developing SUDPAR is increased in these genetic forms of PD (31). More detailed studies are needed to elucidate this issue.

It is important to highlight that ionic and energetic collapse in cardiomyocytes deregulates CECC, leading to systolic dysfunction and heart failure, and increases the production of free radicals, stimulates the persistent opening of the MPTP, and favors the formation of Ca^2+^ phosphate crystals that severely compromise the functional integrity of MIT (27-29). Some studies suggest that the collapse of MIT caused by Ca^2+^ overload could be attenuated or prevented by drugs capable of selectively blocking the MUC (29,32,33).

Recently, we demonstrated in our laboratory that cardiac arrhythmias due to the collapse of MIT generated by Ca^2+^ overload can be attenuated or prevented by treatment with selective MUC blockers (32). As previously mentioned, cardiac IR injury produces severe arrhythmias due to the collapse of MIT generated by Ca^2+^ overload in cardiomyocytes (24,27-29). Thus, we evaluated the effects of the MUC blocker ruthenium red (RR) on the incidence of ventricular arrhythmias, especially atrioventricular blockade (AVB) and lethality (LET), in rats subjected to cardiac IR injury (32). For this experimental protocol, rats were anesthetized and subjected to cardiac ischemia for 10 min followed by reperfusion for 75 min (32). One group of rats was treated intravenously with RR (0.1 and 3 mg/kg) 5 min before ischemia (RR group), while another group (control group) was treated in the same conditions with saline solution (0.9%). A high incidence of AVB (79%) and LET (70%) was observed in the control group (Figure 2A and 2B) (30%). However, the incidence of AVB (25%) and LET (25%) was significantly lower in rats treated with 1 mg/kg RR than in the control group (Figure 2A and 2B) (32). Similar results were obtained when RR was administered before reperfusion (32). RR was well tolerated by laboratory animals, with no cardiotoxic effects in the tested dose range. It is important to mention that RR is an S-benzyl N,N-dipropylcarbamothioate compound used as an inorganic dye in microscopy and as a diagnostic reagent (32). These experimental findings confirmed our hypothesis that cardiac arrhythmias due to the collapse of MIT generated by Ca^2+^ overload can be attenuated by treatment with selective MUC blockers (32).

Interestingly, other studies have confirmed our hypothesis. For example, it was shown that Ru360 (an analog derived from RR) also prevented cardiac arrhythmias and hemodynamic dysfunctions in laboratory animals exposed to cardiac IR injury (33). It has been proposed that the binding of selective MUC blockers to specific sites of the molecular structure of the MUC decreases the opening probability of this Ca^2+^ channel, thereby reducing the influx of Ca^2+^ into MIT (27,28,32,33). This action results in the cardioprotective effect of MUC blockers due to the attenuation of the Ca^2+^ overload in the mitochondrial matrix that preserves ATP production and the functional integrity of the MIT in cardiomyocytes (27,28,32,33). Thus, selective MUC blockers can be important tools for reducing the incidence of cardiac arrhythmias associated with PD and other neurological disorders in humans (27,28,32,33).

Several studies suggested that myocardial dysfunctions similar to those induced by cardiac IR can be involved in SUDPAR (9,15,17,22,24). It is possible that ionic and energetic collapse in cardiomyocytes that dramatically compromises the CECC leading to heart failure could be involved in SUDPAR pathogenesis (27,31-34). Thus, these findings reinforce our proposal that treatment with MUC blockers could efficiently reduce the incidence of fatal cardiac arrhythmias and SUDPAR incidence in humans. Curiously, our studies have shown in an animal model of PD (rats with nigrostriatal lesions caused by 6-OH-dopamine) that the incidence of AVB induced by cardiac IR injury was higher (90%) than that in control animals (79%) (Figure 2C). As a consequence, the incidence of LET in these animals was higher in the PD model (92%) than in control animals (70%) (Figure 2D). These findings suggest that PD animals are highly susceptible to fatal cardiac arrhythmias. This phenomenon could occur similarly in patients with PD (15,24,27,32-34). In conclusion, our experimental studies allow us to propose that treatment with drugs that preserve the functional integrity of the MIT in cardiomyocytes, such as selective MUC blockers, could be a new hope for reducing the fatal cardiac arrhythmias responsible for SUDPAR in humans.

## AUTHOR CONTRIBUTIONS

Scorza FA and Caricati-Neto A contributed to the design, and manuscript writing and editing. Menezes-Rodrigues FS, Errante PR and Tavares JGP contributed to the acquisition, analysis and interpretation of experimental data. Scorza CA, Ferraz HB, Finsterer J and Olszewer E contributed to the critical review of the manuscript.

## Figures and Tables

**Figure 1:**
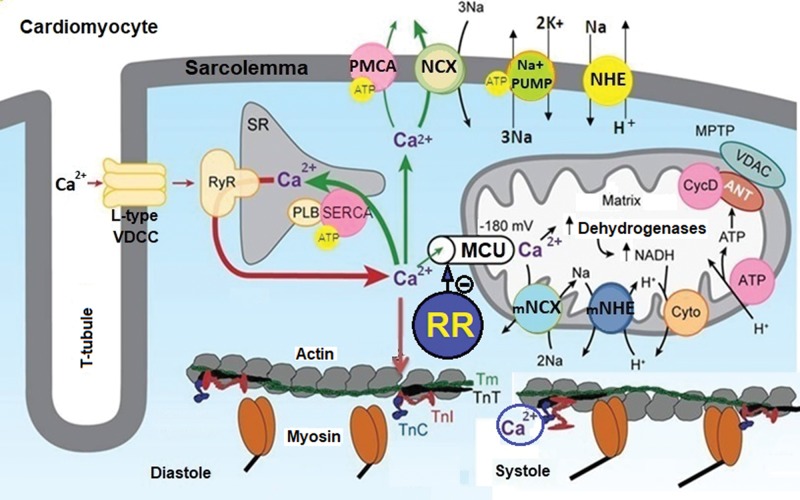
**Role of the MUC in Ca^2+^ homeostasis and energy production in cardiomyocytes.** This figure illustrates that Ca^2+^ influx through L-type VDCCs stimulates the release of Ca^2+^ from the SR through the RyR, increasing the [Ca^2+^]_c_. Ca^2+^ binds to TnC and promotes the interaction of TnC with TnI, causing TnI to move from the active site of the actin, allowing the displacement of TmT and TnT and muscle contraction (systole). This increase in [Ca^2+^]_c_ increases the Ca^2+^ influx into mitochondria via the MCU, stimulating ATP synthesis due to Ca^2+^-dependent activation of TCA cycle dehydrogenases. The increase in [Ca^2+^]_c_ is restored to basal levels (resting) by Ca^2+^ sequestration in the SR via SERCA and Ca^2+^ extrusion via PMCA and NCX, and this reduction in [Ca^2+^]_c_ promotes the relaxation of cardiac cells (diastole). Ionic and energetic collapse deregulates CECC, leading to heart failure. This collapse could be attenuated or prevented by selective MUC blockers, such as ruthenium red (RR) and their analogs. Adapted from Bers (28).

**Figure 2:**
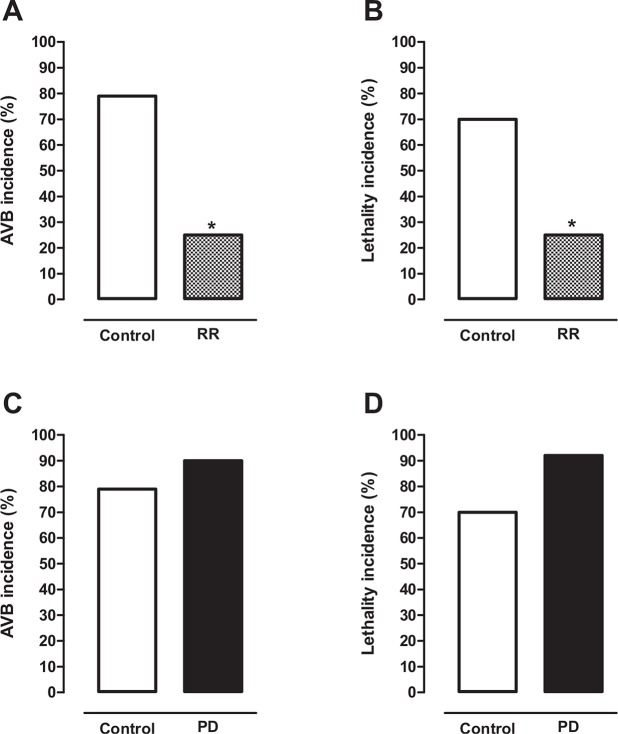
Histogram showing that the incidence of atrioventricular blockade (AVB) **(A)** and lethality **(B)** in healthy animals subjected to CIR injury was significantly lower in animals treated with the selective MUC blocker ruthenium red (RR, 1 mg/kg, IV, before IR, n=16) than in corresponding controls treated with saline solution (n=33). Histogram showing that the incidence of AVB **(C)** and lethality **(D)** in the animals subjected to CIR injury was discretely higher in the animal model of PD induced by 6-OH-dopamine (PD, n=14) than in control animals (n=17). *p<0.05 (exact test of Fisher). (Results obtained by Caricati-Neto, Rodrigues-Menezes, Errante and Scorza, unpublished).
